# Exploring the molecular mechanisms and shared gene signatures between rheumatoid arthritis and diffuse large B cell lymphoma

**DOI:** 10.3389/fimmu.2022.1036239

**Published:** 2022-10-31

**Authors:** Haoguang Li, Le Yu, Xiuling Zhang, Jingjing Shang, Xinwang Duan

**Affiliations:** Department of Rheumatology and Immunology, The Second Affiliated Hospital of Nanchang University, Nanchang, Jiangxi, China

**Keywords:** rheumatoid arthritis (RA), diffuse large B-cell lymphoma (DLBCL), WGCNA, immune response, LGALS2

## Abstract

The relationship between rheumatoid arthritis (RA) and diffuse large B-cell lymphoma (DLBCL) is well characterized, but the molecular mechanisms underlying this association have not been clearly investigated. Our study aimed to identify shared gene signatures and molecular mechanisms between RA and DLBCL. We selected multiple Gene Expression Omnibus (GEO) datasets (GSE93272, GSE83632, GSE12453, GSE1919) to obtain gene expression levels and clinical information about patients with RA and DLBCL. Weighted gene co-expression network analysis (WGCNA) was used to research co-expression networks associated with RA and DLBCL. Subsequently, we performed enrichment analysis of shared genes and screened the most significant core genes. We observed expression of the screened target gene, galectin 2 (*LGALS2*), in DLBCL patients and its impact on patient prognosis. Finally, we analyzed the molecular functional mechanism of LGALS2 and observed its relationship with the immune response in DLBCL using single-sample Gene Set Enrichment Analysis (ssGSEA). WGCNA recognized two major modules for RA and DLBCL, respectively. Shared genes (551) were identified for RA and DLBCL by observing the intersection. In addition, a critical shared gene, *LGALS2*, was acquired in the validation tests. Next, we found that the expression level of *LGALS2* gradually decreased with tumor progression in DLBCL and that increased expression of *LGALS2* predicted a better prognosis for DLBCL patients. ssGSEA revealed that *LGALS2* is involved in immune-related pathways and has a significant regulatory effect on human immune responses. Additionally, we observed that *LGALS2* is closely related to the sensitivity of multiple chemotherapeutic drugs. There is extremely little research on the molecular mechanism of correlation between RA and DLBCL. Our study identified that LGALS2 is a potential therapeutic target and an immune-related biomarker for patients with RA and DLBCL.

## Introduction

Rheumatoid arthritis (RA) is a chronic systemic disease characterized by inflammatory synovitis, predominantly affecting the small joints of the hand and foot, which can lead to joint deformity and dysfunction. RA mainly occurs in middle-aged women with a peak age of onset in the 40s and 60s, often accompanied by a positive serum rheumatoid factor. The worldwide prevalence of RA is reported to be 0.5–1% (1). The pathogenesis of RA is incompletely understood. The interaction between genetic and environmental factors may lead to immune dysfunction, in which various innate immune cells, including mast cells, dendritic cells, neutrophils, macrophages, and natural killer (NK) cells, and components of the adaptive immune system, such as B and T lymphocytes, play an important role. This dysfunction prompts the production of many inflammatory factors and autoantibodies, causing damage to tissue and organs (2).

The relationship between RA and cancer has received much research attention, which is an important factor that seriously affects the already compromised quality of life and survival of RA patients, posing a challenge to clinical management (3). Previous research suggests that the risk of lymphoma, particularly diffuse large B cell lymphoma (DLBCL), is significantly elevated in RA patients (4–6), suggesting that some susceptibility factors in RA may trigger the onset and development of DLBCL. At present, it is widely accepted that persistent chronic inflammation, overactivation of B cells, and genetic susceptibility may provide an appropriate condition for the occurrence of DLBCL in RA patients (7). In addition, as an autoimmune disease, RA requires long-term immunosuppressive treatment, which makes it complicated for many studies investigating the relationship between RA and lymphoma to separate the effects of disease-modifying anti-rheumatic drugs (DMARDs) such as methotrexate from the effects of the RA itself. Therefore, it is not difficult to understand the inconsistent conclusions reached by many studies, with several studies reporting the effect of DMARDs on the development of DLBCL (8, 9), whereas others did not (10–12). Nonetheless, in previous research, the genomic profile of DLBCL with numerous markers potentially relevant for pathogenesis of RA have been identified (7, 13), implying co-inherited risk factors in malignant lymphomas emerging in the context of RA. However, until now, few studies have explored the molecular mechanism of correlation between RA and DLBCL based on bioinformatics analysis, and the common pathogenesis of RA and DLBCL is unclear. Due to the high incidence of DLBCL in RA with insidious in onset and poor prognosis, early screening, early diagnosis and effective treatment are particularly important. Consequently, investigating the mechanisms by which autoimmunity contributes to the development of DLBCL may provide unique insights into the complicated events underlying lymphomagenesis, facilitating the identification of potential diagnostic and prognostic biomarkers and therapeutic targets.

With rapid advances in gene microarray technology, researchers can measure the expression levels of many thousands of genes in a short period, which aids in the deeper understanding of the pathogenesis of diseases at the genetic level. In the present study, we employed bioinformatics analysis to identify common pathways, core shared genes, and core genes involved in the pathogenesis of RA and DLBCL, aiming to explore common mechanisms and therapeutic targets in RA and DLBCL, which will help better manage RA patients and provide early diagnosis and treatment for DLBCL.

## Materials and methods

### Data download and processing

In order to obtain suitable sequencing data, we screened the Gene Expression Omnibus (GEO) database for transcriptome sequencing datasets associated with Rheumatoid Arthritis (GSE) or Diffuse Large B-cell Lymphoma (GSE). Complete information on both datasets can be downloaded from the GEO database. Gene mutation information, patient clinical information, and genome-wide transcript levels of patients with DLBCL were obtained from The Cancer Genome Atlas (TCGA) database (https://portal.gdc.cancer.gov/). In addition, we acquired a list of all genes associated with the immune response from the InnateDB database (https://www.innatedb.com/). In our study, we applied background correction and normalization for all raw data and matched all probe names to their corresponding gene symbols, which were then used in subsequent analyses.

### Weighted gene co-expression network analysis

With the development of bioinformatics, more scientific and comprehensive algorithms are emerging in an endless stream. WGCNA is one of the most popular algorithms for computing large volumes of data, which may be able to filter a series of genes that are most closely related to disease development through clustering and modularization (14). In our study, through the R package “WGCNA”, we built a gene co-expression network of RA and DLBCL.

### Identification and features of shared genes in RA and DLBCL

We selected the intersection of genes in each clinically relevant module as a plausible shared gene. Next, we used the data from TCGA to validate the relationship between these shared genes and RA and DLBCL, and to observe the location of these genes on chromosomes and copy number variation (CNV) frequency. The “ClueGO” and “MCODE” plugs in Cytoscape allowed us to conduct GO enrichment analysis of shared genes to better understand their functions and build Protein–Protein Interaction (PPI) networks, which we used to identify the relationships between proteins and may be instrumental in screening the core genes that play the most important roles. In our study, PPI construction was mainly performed using the STRING database (https://string-db.org/), which is designed to consolidate all available and predicted connections between proteins (15).

### Functional enrichment analysis and gene set enrichment analysis

Gene Ontology (GO)/Kyoto Encyclopedia of Genes and Genomes (KEGG) enrichment analysis is the most universally used and comprehensive functional enrichment method in current medical research. We applied the “clusterProfiler” package in R to accomplish GO/KEGG enrichment analysis of shared genes, which may clarify potential mechanisms of disease occurrence and development. GSEA is a more reliable enrichment method based on the level of gene expression. In our research, taking the median expression level of the *LGALS2* gene as the classification standard, we divided DLBCL patients into low and high expression groups. Thereafter, we performed GSEA enrichment analysis on the two groups of genes using GSEA software (the h.all.v7.2.symbols.gmt gene set as a reference).

### Association between core genes and DLBCL

We used TCGA and GTEx databases of gene expression profiles in DLBCL and normal tissues to compare the differential expression of core genes in DLBCL. Next, we observed the correlation between core genes and clinicopathological staging of DLBCL in TCGA. As we all know, clinicopathological staging is closely related to patient prognosis. As a result, we investigated the impact of core gene expression on the prognosis of DLBCL patients by analyzing the clinical information of patients in TCGA database. Moreover, the risk model built by multivariate Cox regression analyses enables a more comprehensive assessment of the potential impact of core gene expression on patient prognosis. We used the “randomForestSRC” package of R to complete the multivariate Cox regression analyses.

### Assessment of the immune landscape

As research into disease progresses, it becomes clear that the impact of the immune system response on disease progression cannot be ignored (16). The infiltration of immune cells in tumor tissue has a significant impact on tumor development (17). First, we used immune cell infiltration and gene expression data from the TIMER database to identify relationships between the expression of core genes and immune cell abundance in DLBCL, and plotted heatmap and bubble plots to show these results (18). Second, we used single-sample Gene Set Enrichment Analysis (ssGSEA), an emerging gene enrichment method, to compare EstimateScore, ImmuneScore, and StromalScore between high and low expression groups of core genes for each sample. In this calculation, we used the R package “GSVA” (gene set variation analysis) to transform the expression matrix of individual genes into an expression matrix for a specific set of genes (19). Third, we used Spearman’s correlation analysis to analyze the relationship between core genes and a range of immune-related genes, such as immune checkpoint-associated genes and immune cell subpopulation-associated genes.

### DNA stemness score and RNA stemness score

Cancer stemness is one of the most distinctive features of tumors (20). Therefore, Tathiane et al. (20) developed a new computational method, DNAss based on DNA methylation and RNAss based on mRNA expression, to assess the relationship between genes and cancer stemness. In this analysis, we scored according to the following criteria: 1 for the highest degree of differentiation and 0 for no differentiation.

### Statistical analysis

All analyses and visualization were performed in R software (version.4.0.5). Unless otherwise stated, all *t*-tests in this study used *P*<0.05, |logFold Change|>2 as the criteria for statistical significance.

## Results

### Selected datasets

We selected GSE93272 and GSE83632 from the GEO database as the relevant datasets for RA and DLBCL, respectively. A total of 275 whole blood samples were collected in GSE93272, including 245 from patients with RA and 30 from healthy individuals as controls. Similarly, GSE83632 contains 76 samples from patients with DLBCL and 87 samples from healthy donors. In addition, we selected GSE12453 (including 11 blood samples from patients with DLBCL) and GSE1919 (containing five synovium samples from patients with RA and five from normal donors) as validation datasets.

### Co-expression modules in RA and DLBCL

**Figure 1:**
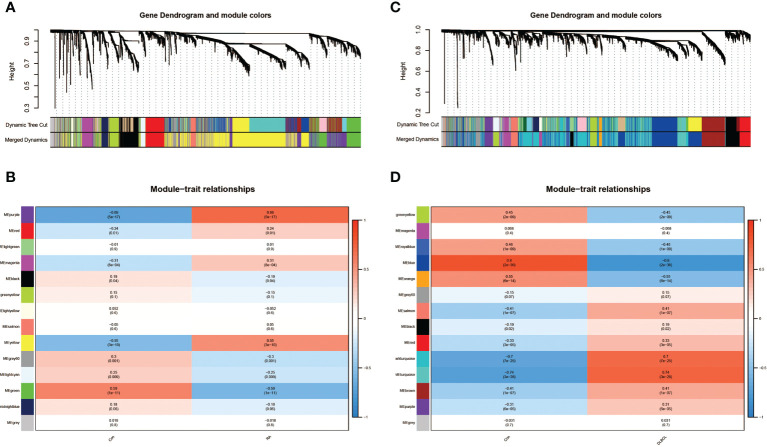
Identification of modules linked to clinical features of RA and DLBCL using WGCNA. **(A)** Cluster dendrogram of co-expressed genes in RA; **(B)** Heatmap of module–trait relationships in RA; **(C)** Cluster dendrogram of co-expressed genes in DLBCL; **(D)** Heatmap of module–trait relationships in DLBCL. RA, rheumatoid arthritis; DLBCL, diffuse large B-cell lymphoma; WGCNA, weighted gene co-expression network analysis.

By performing WGCNA analysis on dataset GSE93272, we recognized 14 modules of genes closely related to the occurrence of RA compared to normal blood samples, each of which was marked by a different color. Among them, we found that four modules, “purple”, “red”, “magenta”, and “yellow”, displayed extremely positive connections with RA (purple module: *r*=0.69, *P*=5e-17; red module: *r*=0.24, *P*=0.01; magenta module: *r*=0.31, *P*=8e-04; yellow module: *r*=0.55, *P*=3e-10; **Figures 1A, B**). In addition, we also identified 14 modules in the DLBCL dataset GSE83632, but eight of these showed a significant positive regulatory relationship with DLBCL (dark turquoise module: *r*=0.7, *P*=7e-25; turquoise module: *r*=0.74, *P*=3e-28; salmon module: *r*=0.41, *P*=1e-07; brown module: *r*=0.41, *P*=1e-07; **Figures 1C, D**). Taking into account the number of genes and the degree of correlation, we finally selected the RA-related purple module and yellow module and the DLBCL-related dark turquoise module and turquoise module as the target modules respectively.

### Shared genes in RA and DLBCL

**Figure 2:**
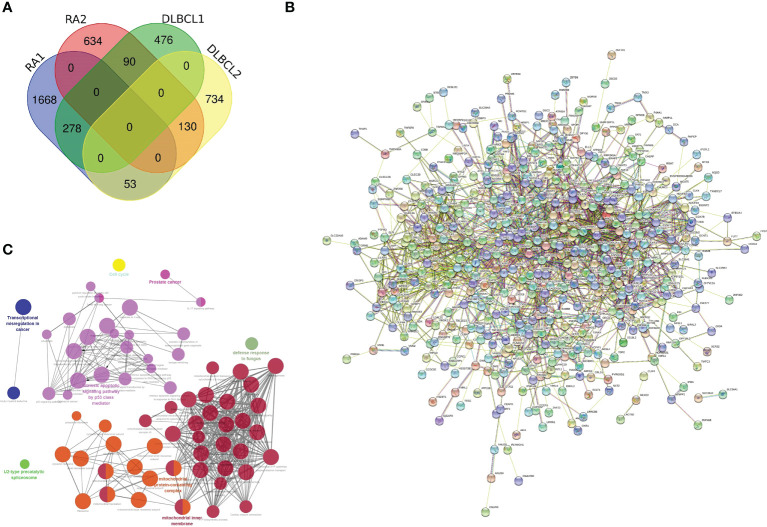
Characterization of the shared genes in RA and DLBCL. **(A)** Venn diagram of the shared genes between the two RA modules and two DLBCL modules; **(B)** PPI network of 551 shared genes; **(C)** The network of GO terms in ClueGO. RA, rheumatoid arthritis; DLBCL, diffuse large B-cell lymphoma; PPI, protein-protein interaction; GO, Gene Ontology.

**Figure 3:**
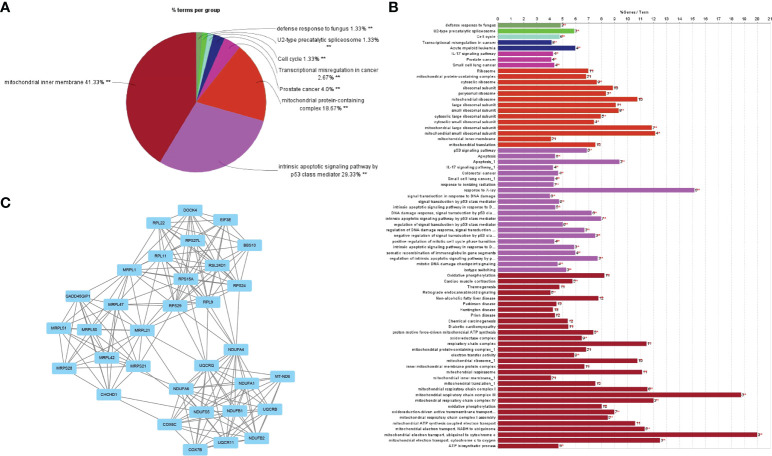
GO analysis of the shared genes in RA and DLBCL. **(A)** The percentage of GO terms in the shared genes, **P < 0.01; **(B)** GO biological process of the four clusters; **(C)** PPI network of 128 most significant shared genes. RA, rheumatoid arthritis; DLBCL, diffuse large B-cell lymphoma; GO, Gene Ontology; PPI, protein-protein interaction.

After noting the intersection of the target module genes selected in the previous step, we obtained 551 genes (**Supplementary Data S1**) involved in the regulatory processes of both RA and DLBCL (**Figure 2A**). Next, using the interaction relationship between these 551 genes, we constructed a PPI network (**Figure 2B**), imported this network into Cytoscape, and used the “MCODE” plugin to filter genes with linkage numbers of more than 20. In total, 128 genes were finalized as the most significant shared genes (**Figure 2C**). Using results from “ClueGO” analysis, we identified the potential mechanisms of these 128 shared genes in RA and DLBCL. We found that these genes were enriched in biological activities, such as intrinsic apoptotic signaling pathway by p53 class mediator, mitochondrial protein-containing complex, mitochondrial inner membrane, and transcriptional misregulation in cancer (**Figures 3A-C**).

### *LGALS2* is the core shared gene

**Figure 4:**
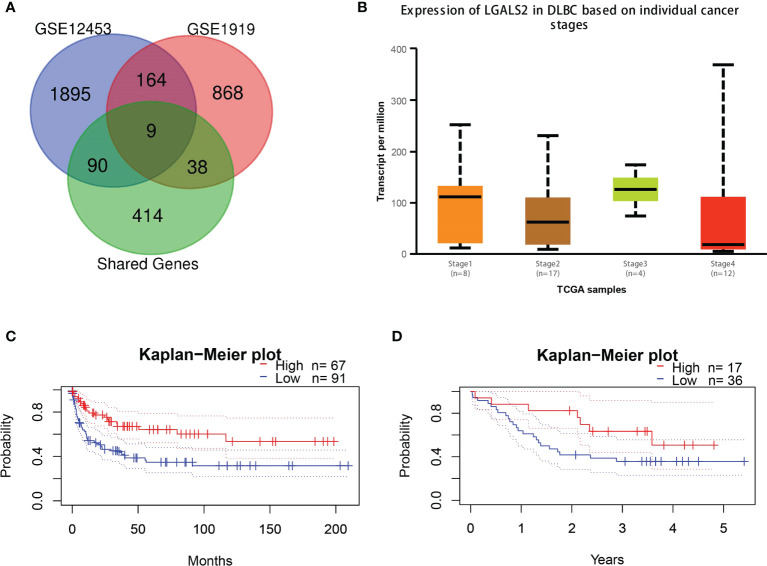
*LGALS2* expression and prognostic value in DLBCL tissues. **(A)** Venn diagram of the shared genes in RA and DLBCL in validation cohorts; **(B)** The expression of *LGALS2* in DLBCL at different pathological stages; **(C, D)** Kaplan–Meier curve of association of *LGALS2* and DLBCL patients’ OS in GSE4475 **(C)** and E-TABM-346 **(D)**. RA, rheumatoid arthritis; DLBCL, diffuse large B-cell lymphoma; OS, overall survival.

To ensure the accuracy of the screened shared genes, we applied the differential genes in GSE12453 and GSE1919 for validation. By intersecting the shared genes with genes in the two datasets, we obtained nine shared genes (*GMFG*, *BCL2A1*, *LGALS2*, *TNFSF8*, *SLC39A8*, *VAMP8*, *MTHFD2*, *GZMA*, *CD69*) with high reliability (**Figure 4A**). Considering the expression and prognosis of genes in DLBCL, we finally determined that *LGALS2* is the core shared gene between RA and DLBCL. The *LGALS2* expression level gradually decreased with the deterioration of DLBCL (**Figure 4B**). In addition, we found that DLBCL patients with high expression of *LGALS2* may demonstrate longer postoperative survival periods (**Figures 4C, D**).

### Biological processes associated with LGALS2 and LGALS2-related signaling pathways

**Figure 5:**
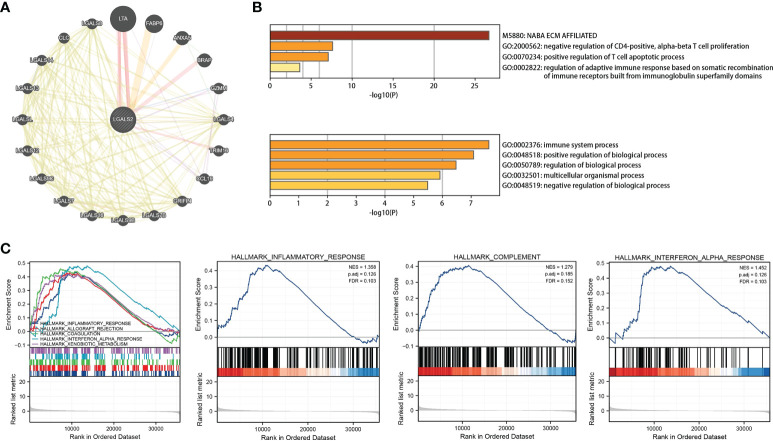
PPI network and functional enrichment analysis of *LGALS2*. **(A)** PPI network of LGALS2 and its interacting proteins; **(B)** GO enrichment analysis of *LGALS2* and its interacting proteins; **(C)** GSEA of the top 10 enriched pathways in DLBCL patients with high *LGALS2* expression.

After identifying *LGALS2* as the core gene in DLBCL, we next explored the mechanisms underlying *LGALS2* functions using the GeneMANIA database (http://genemania.org/), an online platform used to search for proteins associated with specific genes and gene sets (21). We constructed the PPI network for *LGALS2* and determined the proteins related to *LGALS2* (**Figure 5A**). Thereafter, we performed GO/KEGG functional enrichment analysis and GSEA enrichment analysis on these genes, and the results showed that *LGALS2* was related with NABA_ECM_AFFILIATED, negative regulation of CD4-positive, alpha-beta T cell proliferation, positive regulation of T cell apoptotic process, regulation of adaptive immune response based on somatic recombination of immune receptors built from immunoglobulin superfamily domains, and immune system process (**Figure 5B**), as well as inflammatory, complement, and interferon alpha response (**Figure 5C**).

### Association between LGALS2 and tumor immune microenvironment

**Figure 6:**
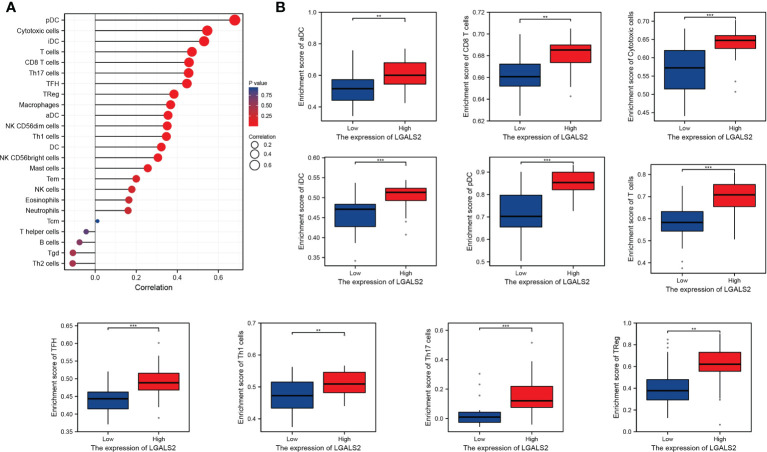
Distribution of immune cell infiltration in DLBCL. **(A)** Relationship of LGALS2 expression and immune cell subtypes in DLBCL patients; **(B)** Bar plot of LGALS2 expression and immune cell subtypes. **P < 0.01, ***P < 0.001.

**Figure 7:**
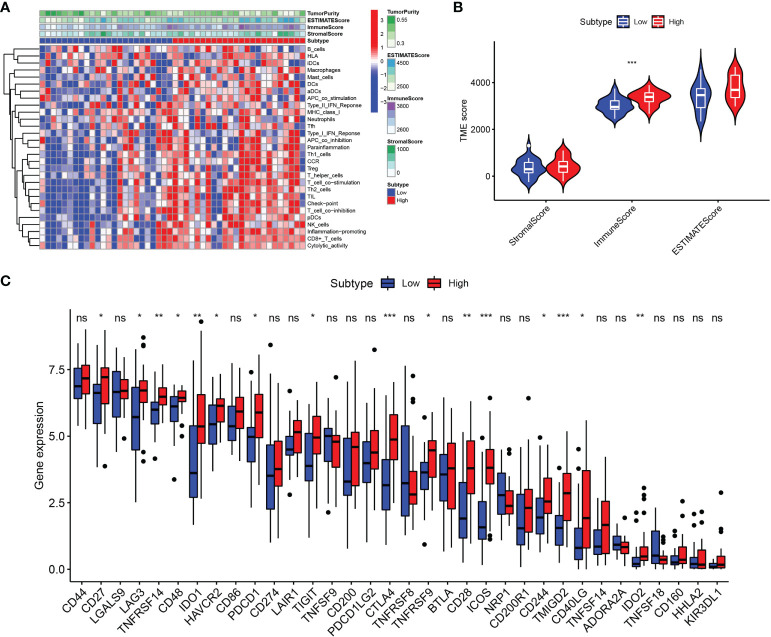
Immune microenvironment analysis in DLBCL patients with high and low LGALS2 expression. **(A)** Heatmap of the immune cells between high and low expression groups; **(B)** Comparison of ESTIMATE Score, Stromal score, and Immune score between LGALS2-high and LGALS2-low groups using the ssGSEA algorithm; **(C)** immune checkpoints between LGALS2-high and LGALS2-low groups. ssGSEA, single-sample Gene Set Enrichment Analysis. ns, no significance, *P < 0.05, **P < 0.01, and ***P < 0.001.

Based on the ssGSEA algorithm, we derived the relationship between the expression of LGALS2 and the infiltration of immune cells in DLBCL (**Figures 6A, B**). The results showed that the expression of LGALS2 was positively associated with plasmacytoid dendritic cells (pDCs), Cytotoxic cells, interdigitating cells (iDCs), T cells, clusters of differentiation 8 (CD8) T cells, T helper 17 (Th17) cells, T follicular helper (TFH) cells, regulatory T (Treg) cells, and macrophages. On the contrary, the expression of LGALS2 was negatively linked with B cells, T gamma delta (Tgd), and T helper 2 (Th2) cells. Thereafter, we calculated the StromalScore, ImmuneScore, and ESTIMATEScore of the two groups with high and low expression of LGALS2. Consistent with the above results, the ImmuneScore of the LGALS2 high expression group was significantly higher than that of the low expression group (**Figures 7A, B**). Finally, immune checkpoint correlation analysis showed that LGALS2 was closely associated with various immune checkpoint factors, such as IDO1, CTLA4, CD28, ICOS, IDO2, and TMIGD2 (**Figure 7C**).

### Drug sensitivity analysis of LGALS2

**Figure 8:**
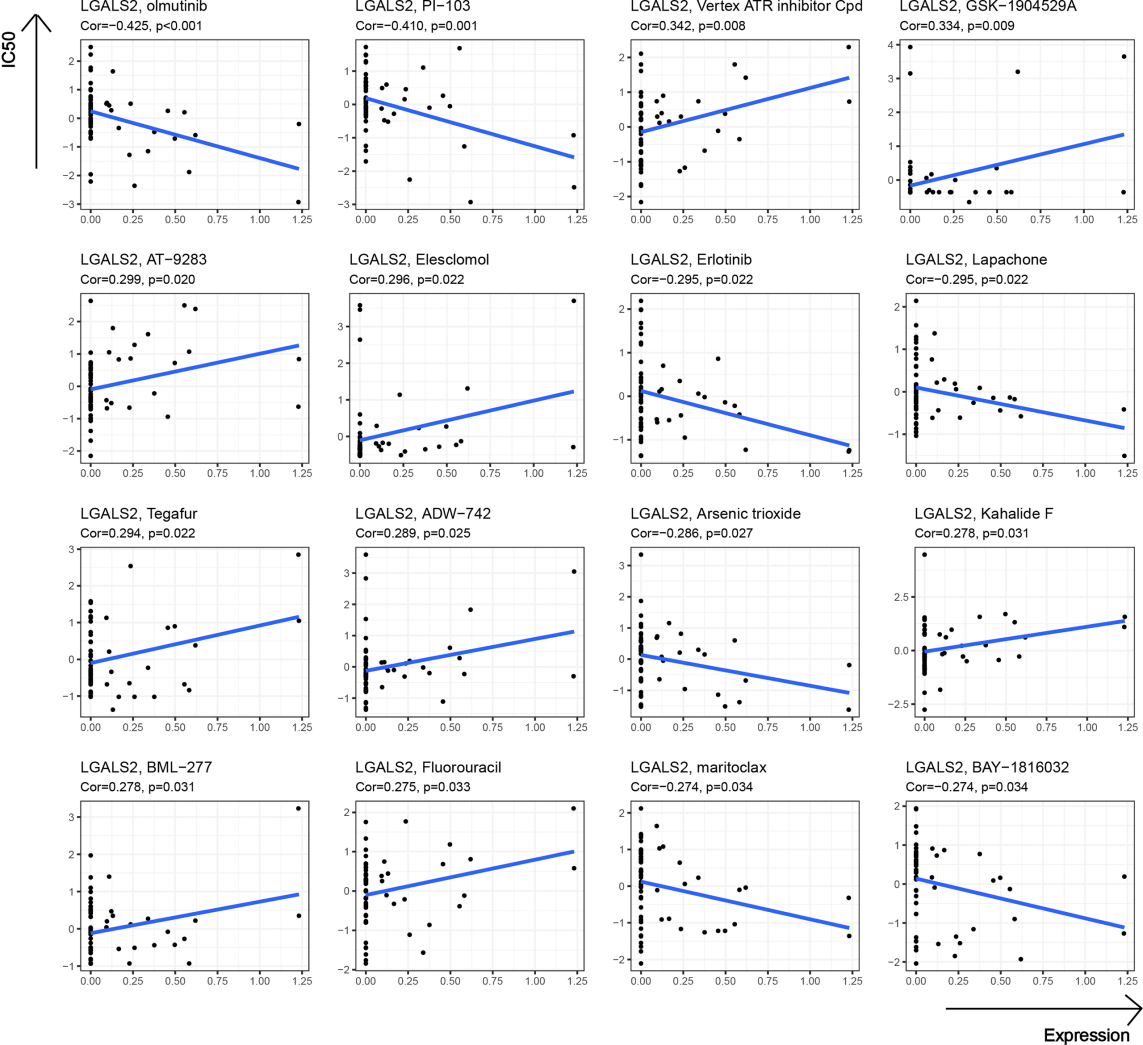
Drug sensitivity analysis of LGALS2.

At the end of our study, we further explored the potential link between drug sensitivity and the expression of LGALS2 using the CellMiner™ database (https://discover.nci.nih.gov/cellminer/home.do), a web-based suite of genomic and pharmacologic tools (22). Notably, LGALS2 expression was negatively related to sensitivity to olmutinib, PI-103, Erlotinib, Lapachone, Arsenic trioxide, maritoclax, and BAY-1816032 while being positively correlated with sensitivity to Vertex ATR inhibitor Cpd, GSK-1904529A, AT-9283, Elesclomol, Tegafur, ADW-742, Kahalide F, BML-277, and Fluorouracil (**Figure 8**).

## Discussion

Rheumatoid arthritis, as one of the most frequent chronic diseases, brings suffering and inconvenience to the lives of more than 40 million people worldwide (23). Although RA itself does not pose a threat to patients’ lives, a series of complications in RA patients can seriously affect their quality of life and mortality. However, it is troublesome that the pathogenesis of RA is still undefined. As early as the 1970s, HA et al. (24) found that patients with RA had a higher risk of hematological malignancies, including non-Hodgkin’s lymphoma (NHL), through large volume epidemiological statistics. NHL is the most common lymphatic malignancy, accounting for approximately 80%–90% of all lymphomas (25), of which about 31% are DLBCL (26). DLBCL is often not diagnosed early and easily metastasizes to distant sites. Although the current cure rate of DLBCL is relatively satisfactory, the characteristics of DLBCL make it a serious threat to patients’ lives. Nevertheless, there are sufficient clinical data to demonstrate the association between RA and DLBCL, and previous studies have explored the genomic profile associated with RA and DLBCL (7, 13), but the molecular mechanisms underlying this association have not been clearly investigated. To the best of our knowledge, our study is the first to use a systemic bioinformatic analysis approach to address the mechanism underlying the association between RA and DLBCL.

In our study, we performed co-expression clustering analysis on typical GEO datasets of RA (GSE93272) and DLBCL (GSE83632) using the current most reliable algorithm for WGCNA. Subsequently, we selected the intersection of the two most closely related modular genes. These intersecting genes are the shared genes that we believe may be involved in both diseases. Simultaneously, we observed the biological processes and signaling pathways in which these shared genes are jointly involved. Interestingly, enrichment analysis results contained multiple mitochondrial and apoptosis-related biological processes, which are closely associated with disease progression in RA (27) and DLBCL (28). This result suggested that the development of DLBCL in RA patients was likely due to altered transcription and apoptosis mediated by abnormal mitochondrial function. Interestingly, the results of the functional enrichment analysis also included a biological process, intrinsic apoptotic signaling pathway by p53 class mediator, which is consistent with recent advances in DLBCL. This research suggests that p53 regulates tumor progression in DLBCL (29). In addition, we screened for key genes with higher reliability from the 551 shared genes and found nine core genes most likely to mediate RA and DLBCL occurrence by taking intersections with the validation dataset GSE12453 and GSE1919. We finally identified our target gene as *LGALS2*.

Galectins (LGALS) is a protein-coding family containing 15 members, which encode proteins that bind carbohydrates and beta-galactoside and regulate transcriptional processes (30). LGALS family members contain two conserved structural domains: a domain that binds to the lactosamine unit within the glycan and a carbohydrate recognition domain (CRD) consisting of 130 amino acids (31). *LGALS2*, a member of this family, encodes a protein that is abundantly expressed in various mammalian cells and is involved in immunomodulatory effects by regulating LGALS2 expression in different inflammatory immune cells in a way that induces cell migration, activation, and release of inflammatory factors (32). Furthermore, the regulatory role of LGALS2 in diseases has been well reported in previous studies. For example, LGALS2 was originally studied for its function in cardiovascular diseases as an inflammatory factor (33, 34). Subsequently, since LGALS2 is abundantly expressed in the intestinal epithelium and has been shown to induce apoptosis of T cells, many studies have focused on the functional role of LGALS2 in inflammatory bowel disease (35). In recent years, the role of LGALS2 in cancer development has been explored and it has been proposed as a potential prognostic biomarker for breast cancer (36) and colorectal cancers (37). However, how LGALS2 contributes to diseases and the clear mechanisms performed in the disease need to be discussed with functional studies in the future.

Notably, several studies suggested that genetic polymorphisms in LGALS2 may be strongly associated with susceptibility to RA and related comorbidities (38, 39). Furthermore, we observed the PPI network of LGALS2 and performed functional enrichment analysis. As expected, the results of the functional enrichment analysis focused almost exclusively on the regulation of various immune cells, most of which were T cells, especially CD4 T cells. As an important component of effector T cells, CD4 T cells have been shown to infiltrate the inflamed synovial membrane, and the mediated abnormal immune response of CD4 T cells is considered to be one of the main pathogenic mechanisms of RA (40), supporting the results of this study. Therefore, we speculate that the regulatory effects of LGALS2 on various diseases are likely to be accomplished by modulating the activity of CD4 T cells. Besides, we observed that LGALS2 expression gradually decreased with tumor progression in DLBCL and that high LGALS2 expression predicted a better prognosis for DLBCL patients. Recent studies have reported that chimeric antigen receptor (CAR) T cells are the most promising emerging treatment for DLBCL, particularly effective in the treatment of refractory or recurrent DLBCL (41, 42), which reinforced our suspicions. Nevertheless, there are still limitations to the clinical application of CAR T cell therapy, and our study on the regulation of T cells and DLBCL by LGALS2 may provide insights to solve these limitations. Interestingly, the enrichment analysis of GSEA also showed that LGALS2 was involved in inflammatory response, complement pathway and interferon (IFN) signaling pathway, which were well recognized as important features in the occurrence and development of RA. According to the existing theories and our analysis, the possible mechanism of the susceptibility to DLBCL in RA patients is that LGALS2 causes persistent chronic inflammation and excessive activation of B cells through the production of many inflammatory factors and autoantibodies, thus leading to the occurrence of DLBCL (7), which also supported by previous studies reporting a strong relationship between lymphoma incidence and RA disease severity (43, 44). More importantly, type I IFN is not only one of the molecules involved in RA (45), but also an effective therapeutic target in DLBCL (46). In addition, we also observed the effect of LGALS2 on other immune cells and the relationship with immune checkpoints, the results of which indicated that LGALS2 has a significant regulatory effect on human immune response. Chemotherapy is currently the primary treatment option for DLBCL. Finally, we found that LGALS2 is closely related to the sensitivity of multiple chemotherapeutic drugs. These results indicate that LGALS2 is a meaningful biological target in DLBCL, which is the first discovery reported publicly.

Nevertheless, there are some limitations to our study. Our results remain at the level of data analysis, and there are not enough experimental data to confirm our results. Therefore, reasonable assays should be conducted to verify our conjecture step by step. Taken together, our study explored the molecular mechanism between RA and DLBCL for the first time and identified that LGALS2 is a potential therapeutic target and an immune-related biomarker for patients with RA and DLBCL.

## Data availability statement

The original contributions presented in the study are included in the article/**Supplementary Material**. Further inquiries can be directed to the corresponding author.

## Author contributions

XWD and HGL designed the study. HGL, LY, XLZ, and JJS performed data analysis. HGL drafted the manuscript. XWD revised the manuscript. All authors contributed to the article and approved the submitted version.

## Funding

This study was supported by grants from the Science and Technology Program of Department of Health of Jiangxi Province (20204254), and the Key Research and Development Program of Jiangxi municipal Science and Technology Department (20192BBGL70024). The funders had no role in study design, data collection and analysis, decision to publish, or preparation of the manuscript.

## Acknowledgments

We would like to thank Editage (www.editage.cn) for English language editing.

## Conflict of interest

The authors declare that the research was conducted in the absence of any commercial or financial relationships that could be construed as a potential conflict of interest.

## Publisher’s note

All claims expressed in this article are solely those of the authors and do not necessarily represent those of their affiliated organizations, or those of the publisher, the editors and the reviewers. Any product that may be evaluated in this article, or claim that may be made by its manufacturer, is not guaranteed or endorsed by the publisher.
